# A hydrological simulation dataset of the Upper Colorado River Basin from 1983 to 2019

**DOI:** 10.1038/s41597-022-01123-w

**Published:** 2022-01-20

**Authors:** Hoang Tran, Jun Zhang, Mary Michael O’Neill, Anna Ryken, Laura E. Condon, Reed M. Maxwell

**Affiliations:** 1 Department of Civil and Environmental Engineering, Princeton University, Princeton, NJ USA; 2 Department of Hydrology and Atmospheric Sciences, The University of Arizona, Tucson, AZ USA; 3 NASA/GSFC, Greenbelt, USA; 4 Department of Geology and Geological Engineering, Colorado School of Mines, Golden, CO USA; 5 High Meadows Environmental Institute, Princeton University, Princeton, NJ USA

**Keywords:** Hydrology, Environmental impact

## Abstract

This article presents a hydrological reconstruction of the Upper Colorado River Basin with an hourly temporal resolution, and 1-km spatial resolution from October 1982 to September 2019. The validated dataset includes a suite of hydrologic variables including streamflow, water table depth, snow water equivalent (SWE) and evapotranspiration (ET) simulated by an integrated hydrological model, ParFlow-CLM. The dataset was validated over the period with a combination of point observations and remotely sensed products. These datasets provide a long-term, natural-flow, simulation for one of the most over-allocated basins in the world.

## Abstract (summary)

**Table:** 

Measurement(s)	streamflow • groundwater • evaporation • evapotranspiration • total water storage
Technology Type(s)	integrated hydrological model
Factor Type(s)	temporal interval • geographic location
Sample Characteristic - Environment	river
Sample Characteristic - Location	Colorado River

Machine-accessible metadata file describing the reported data: 10.6084/m9.figshare.16632454

## Background & Summary

The Upper Colorado River Basin (UCRB) is one of the most over-allocated basins in the world. It provides water for 40 million people in Colorado and downstream states of Arizona, California, Nevada, and Utah. However, the UCRB water capacity is decreasing as a combined result of climate change^1–8^ and anthropogenic activities^1,5,9–11^. Understanding the dynamic of the UCRB water cycle is crucial for long-term and short-term water resources management.

While understanding the overallocation of an important headwaters system is challenging and depends on many factors, data (in the form of observations and data products) can help provide insight. There are currently three data sources for studying the UCRB’s water resources, namely, point observations, remote sensing products and model output products. All three are valuable tools to quantify the quantity and flux of water in the system, but each are incomplete.

Point observations, such as the streamflow monitoring network, have existed since the early 20^th^ century in the UCRB. While some observations have dense networks (e.g. there are 630 and 490 monitoring locations for streamflow and air temperature, respectively), others, such as evapotranspiration (ET) and groundwater depth, are more sparse. For example, there is only one AmeriFlux station measuring ET in the entire UCRB (approximately 280,000 km^2^).

Remote sensing products have become a valuable source of earth systems data and are often used on their own or to complement *in-situ* point observations. For example, a family of the Moderate Resolution Imaging Spectroradiometer (MODIS) products has been used globally to study various water cycle components and extremes such as snow^12,13^, flood^14,15^, and drought^16,17^. Remote sensing products’ temporal and spatial resolutions may be limited for some applications. For example, the GRACE remote sensing product have been successfully applied to understand large scale depletion of groundwater worldwide^18^, but its native spatial resolution, at hundreds of kilometers laterally, can be prohibitively coarse for many hydrological applications^19^.

Hydrologic models are also useful tools to understand the quantity and flux of water in the UCRB. A prime example of models used to understand water states and fluxes is the North American Land Data Assimilation System (NLDAS) platform. NLDAS is a collaborative project between NASA, NOAA and a group of the universities and incorporates many different hydrologic models: Mosaic^20,21^, Noah-2.8^22,23^, Soil moisture Accounting Model (SAC^24,25^) and Variable Infiltration Capacity (VIC^26,27^). While useful tools, all hydrologic models simplify some aspects of the hydrologic cycle, demonstrate some mismatch between simulated output and observations, and provide output at a discrete spatial and temporal resolution.

Here we present an integrated hydrologic simulation of UCRB spanning from October 1982 to September 2019 at high spatial (1 km) and temporal (hourly) resolutions. The model that we used, ParFlow-CLM, increases both the number of processes simulated over the UCRB (deep groundwater to the top of the canopy) and the spatial and temporal resolution at which these processes are simulated. To build confidence in the simulation results, ParFlow is exhaustively compared to available observations and data products. While still an imperfect representation of the hydrology of the UCRB, this dataset pushes our modeling capabilities forward and augments existing observations and data products to help provide a more complete understanding of this important watershed.

## Methods

### Site

The Upper Colorado River Basin (UCRB) is a snowmelt-dominated system that covers about 280,000 km^2^. It extends from headwaters in the Rockies in Colorado and Wyoming to Lee’s Ferry in Northern Arizona with elevation ranges between 3300 m and 900 m. During the winter season, from October to the end of April, the snow cover area (SCA) for the UCRB ranges from 50,000 km^2^ to 280,000 km^2^ which plays a crucial role in energy^28^ and hydrological cycles^29^.

### ParFlow-CLM

The hydrologic simulation of the UCRB was conducted using the integrated hydrologic model, ParFlow-CLM^30–32^. ParFlow computes both the surface and subsurface fluxes by solving the Richards equation^33^ in three spatial dimensions together with the kinematic wave equation over a terrain following grid. Furthermore, ParFlow is coupled to a land surface model (Common Land Model; CLM), ParFlow-CLM, to resolve the energy and water balances from the canopy to the ground surface.

The technical details of ParFlow-CLM are well-documented in Maxwell and Miller^30^, Kollet and Maxwell^31,34^, Kollet *et al*.^35^, Maxwell *et al*.^36^, Jefferson and Maxwell^37^, Maxwell and Condon^38^ and Kuffour *et al*.^39^. ParFlow integrates groundwater and surface water systems using a free surface overland flow boundary condition^31^. In other words, the surface water and variably saturated groundwater flow equations directly exchange fluxes without a conductance layer. In ParFlow, streams are formed by either Hortonian (excess infiltration^40^) or Dunne (excess saturation^41^) runoff without the need of a priori embedded rivers.

CLM is the land surface component of the model. CLM solves the terrestrial energy balance (e.g. net radiation, sensible, latent and ground heat fluxes) in addition to a multi-layer snow model^42^ and a complete canopy water balance. Sensible and latent heat are solved through a resistance scheme including soil, vegetation and atmospheric resistances^43^. The ground heat is calculated based on the one-dimensional heat conduction equation^35^. Ground and sensible heat fluxes are directly dependent on the water content in soil layers which is solved by ParFlow^34^. Conversely, soil moisture is also dependent on infiltration and plant uptake which is passed back to ParFlow by CLM^34,37,44^.

### Input datasets

The main inputs in this study can be divided into two groups: dynamic atmospheric forcing and static model parameters. The first group of inputs includes a subset from the North American Land Data Assimilation System (NLDAS) project. The second group of inputs includes two types of model parameters: surface information (i.e. topographic slopes and land cover) and subsurface information (i.e. soil, geology and bedrock types and their characteristics).

The NLDAS project is a collaboration between NASA, NOAA and a group of universities to provide high accuracy and consistent datasets for a wide variety of hydrologic studies. Studies modeling streamflow^45,46^, soil moisture^47,48^ and snow^49,50^ have been using NLDAS as inputs. Thus, we decided to use a subset of the NLDAS dataset for this simulation which includes eight variables, namely, precipitation, air temperature, short-wave radiation, long-wave radiation, east-west wind speed, south-north wind speed, atmospheric pressure and specific humidity. The NLDAS has two versions which were used in this study: NLDAS-1^51,52^ which spans from 1983 to 2002 and NLDAS-2^45,53^ which spans from 2003 to 2019. Major improvements from NLDAS-1 to NLDAS-2 include additional measurement sources of precipitation such as gauge (Climate Prediction Center - CPC product), radar (National Centers for Environmental Prediction-NCEP 4-km hourly Doppler radar Stage II) and satellite (CPC MORPHing technique – CMORPH)^45^.

The model parameters consist of two types: surface and subsurface. The surface parameters, topographic slopes and land cover, were computed as follows. Topographic slopes were calculated using the Priority Flow toolbox^54^ with an elevation input from the Hydrological data and maps based on Shuttle Elevation Derivatives at multiple Scales (HydroSHEDS). Land cover information was obtained from the National Land Cover Database (NLCD) at 30-m resolution. The obtained land cover dataset was then upscaled to model resolution at 1-km. Land cover values are based on the International Geosphere-Biosphere Program (IGBP) classifications.

The subsurface of the ParFlow domain consists of four soil layers at the top and one geology layer at the bottom. Categories for the soil units were obtained from the Soil Survey Geographic Database (SSURGO; https://websoilsurvey.sc.egov.usda.gov) and hydrogeologic categories were obtained from a global permeability map developed by Gleeson *et al*.^55^. Parameters such as saturated hydraulic conductivity and van Genuchten relationships of those soil and hydrogeology layers were obtained from Schaap and Leij^56^. More details about the subsurface parameters and configurations can be found in Condon and Maxwell^57^, and Maxwell *et al*.^36^.

### Model spinup

A model spinup is the initialization process used to bring the system into a more realistic set of initial conditions when the true starting point of the model (for example, the pressures everywhere in the UCRB) is unknown. This starting point is particularly important for groundwater systems which take longer time to evolve than the surface systems.

In preparation for the 37-year simulation, we completed a model spinup in two steps. First, potential recharge (calculated as Precipitation Minus Evapotranspiration (PME)) was applied to the model until the change in subsurface storage was less than 3% of the total storage. The potential recharge PME was derived from the average precipitation and evapotranspiration products for the period between 1950 and 2000 by Maurer *et al*.^58^. For the second step, the hourly atmospheric forcing for the initial water year (1983) was repeatedly applied to bring the model into quasi-equilibrium.

### Simulation from 1983 to 2019

The spinup process described above provided an initial pressure model of the UCRB for the 37-year simulation. To do this, we simulated each year for a time period spanning from October to the end of September next year, often known as the Water Year (WY) which better matches with the precipitation cycle that occurs in late autumn. All simulations were executed on the Cheyenne supercomputer operated by the National Center for Atmospheric Research (NCAR). On average, one WY simulation used about 6,100 cores hours, which resulted in about a day of wall-clock time given parallel computing and batch submission processes. The entire 37-year simulation used approximately 220,000 core hours of computing time, spanning about 1.2 months of wall-clock time.

### Observation datasets used for comparison

A comprehensive comparison between model simulation results, observations and remotely sensed products was conducted. A summary of each dataset is provided below.

Streamflow observations were compiled from the USGS Water Data web service. Since this was a pre-development simulation (i.e. excluding surface water management and groundwater pumping), we filtered out observations from stations that are clearly affected by anthropogenic activities. Although small drainage area basins can have water withdrawals and irrigation ditches, the effect of anthropogenic activities on these basins are much less compared to larger drainage area basins, especially in monthly or annual scales^59,60^. Thus, we defined a drainage area threshold of 500 km^2^; stations whose drainage areas are larger than the threshold were then manually inspected. For example, we removed the station at Lee’s Ferry (drainage area: 289,560 km^2^) located right after the Glen Canyon Dam.

**Fig. 1:**
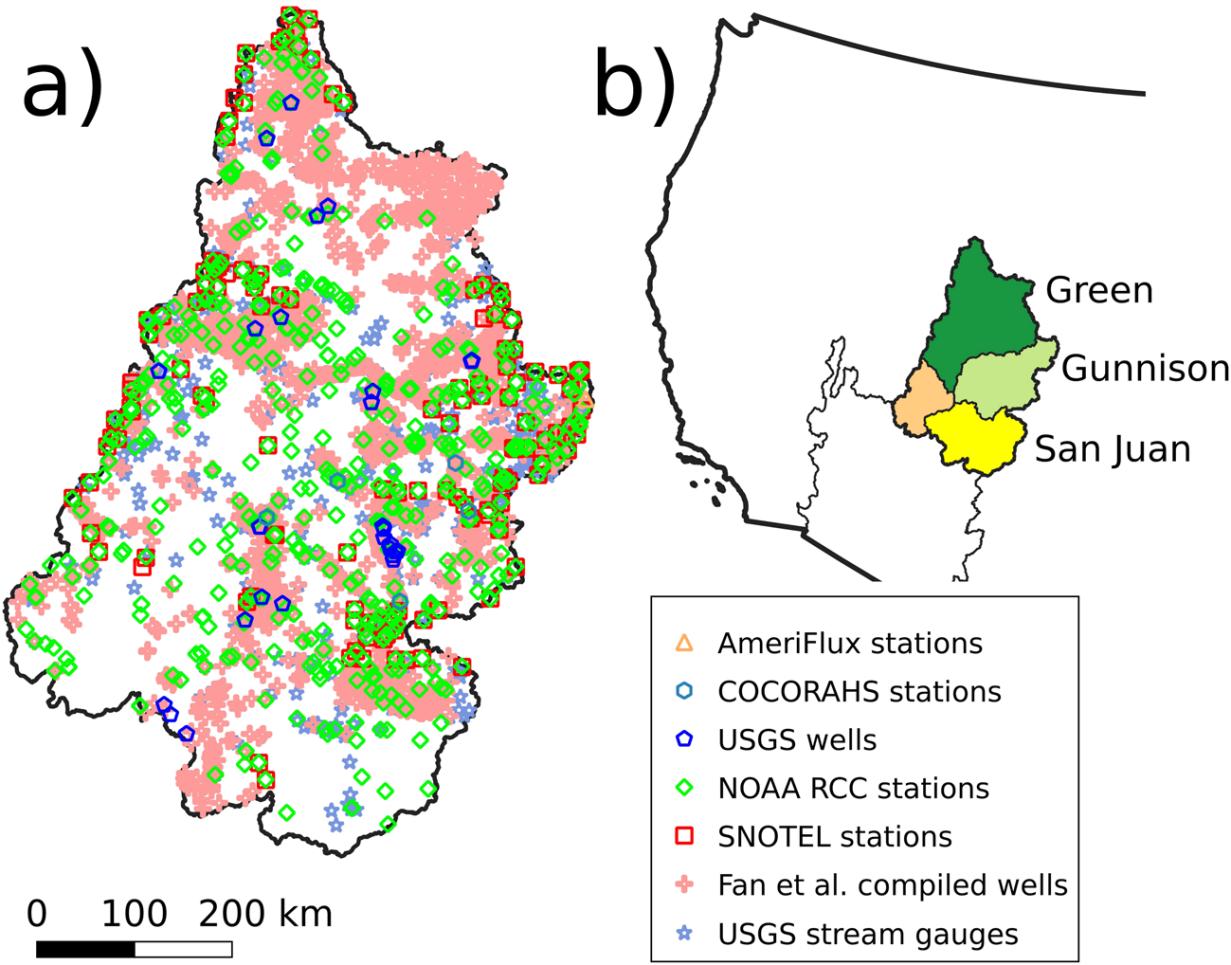
(**a**) Location and type of observations used to compare observations and data products to model simulations, (**b**) Locations of the UCRB and its major sub-basins.

**Fig. 2:**
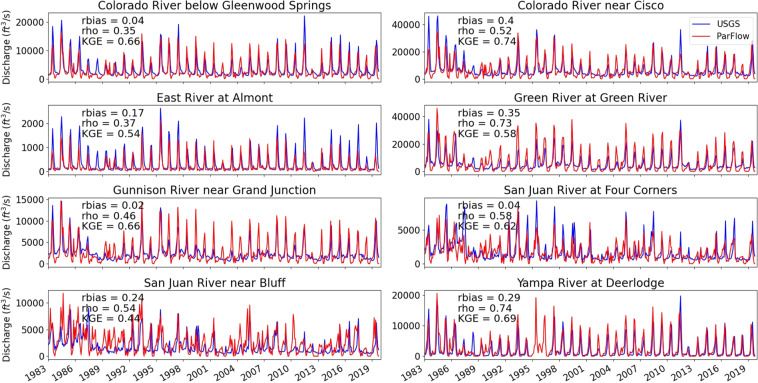
Plots of simulated and observed streamflow for eight gages within the UCRB. Streamflow predicted by ParFlow is shown using the red line while streamflow predicted by the natural flow model is shown in blue.

In total, there were a total of 602 UGSG stream stations in the UCRB with observations from 1983 to 2019 (shown as blue stars in Fig. 1). Eight stations situated at the outlet of watersheds that represent medium to large drainage areas were used for comparison demonstration in Fig. 2. These stations were: Green River at Green River (116,160 km^2^), Colorado River near Cisco (62,419 km^2^), San Juan River near Bluff (59,570 km^2^), Yampa River at Deerlodge (20,541 km^2^), Gunnison River near Grand Junction (20,520 km^2^), Colorado River below Glenwood Springs (15,576 km^2^), San Juan River at Four Corners (37,813 km^2^), and East River at Almont (749 km^2^).

In addition to the USGS stream observations, we also used the Bureau of Reclamation natural flow dataset^61–66^ which is available for 20 stations in the UCRB from 1906 to 2020. The natural flow was constructed by combining history gauge flow with consumptive uses and losses^64^ and reservoir regulation^66^. The dataset has been used in several other studies including drought analysis in the UCRB^67–69^.

The next observational set was the USGS groundwater database (https://waterdata.usgs.gov/nwis/gw). All data from wells that have at least two months of observations during the period between 1983 and 2019 were used for comparison here. Measurements that did not pass the USGS quality control (i.e. flagged for potential measurement inconsistency or negative outlier values) were filtered out. Also, wells with water table depths (WTD) below 52 m (i.e. below the depth for the center of the bottom grid cell in the model domain) were removed. A total of 36 wells were used to compare water table levels after this filtering process (shown as blue hexagons in Fig. 1).

In addition to these temporal groundwater observations, there are a total of 3,865 well locations in the UCRB from the Fan *et al*.^70^ water table observations. Fan *et al*.^70^ compiled this water table observational dataset by calculating the average WTD for USGS sites between 1927 and 2009. While Fan *et al*.^70^ noted that about 90% of the wells have only one observation at different times, they found that wells whose WTD were above 20 m aligned well with their global simulated WTD. Based on these findings, their dataset was determined to be an appropriate resource to validate model performance.

We also employed a derived snow cover extent from MODIS for comparison to simulations. The daily cloud-free snow cover dataset^71^ was developed via a series of mitigated cloud filters and the Variational Interpolation algorithm to the MODIS-Snow Cover Area (SCA) Daily (MOD10C1 and MYD10C1) version 6 product^12,72^. The product has been proved to effectively capture the dynamic changes of snow from 2000 to 2017 with the average of Probability Of Detection and False Alarm Ratio are 0.955 and 0.179, respectively^71^. The cloud-free product’s spatial and temporal resolutions are 0.05° and daily, respectively.

The Snow Water Equivalent (SWE) data was obtained from the Snow Telemetry (SNOTEL) network. There was a total of 133 SNOTEL stations used for comparison. SNOTEL stations have an average elevation of nearly 2,900 m with the station in the highest elevation of more than 3,500 m at the Italian Creek, CO.

Total water storage (TWS) change measured by the Gravity Recovery and Climate Experiment (GRACE) mission was used to compare with simulated TWS. Launched in 2002, GRACE estimates monthly changes in terrestrial water storages globally based satellite location (http://www2.csr.utexas.edu/grace/RL05_mascons.html). The data used in this study, CSR Release-06 GRACE Mascon Solutions, was released from the Center for Space Research (CSR), the University of Texas at Austin. Mass fluxes (measured in terms of mass concentration—mascon) derived directly from raw GRACE data often have north-south stripes due to modeling errors, measurement noise and observability issues^73^. To decrease the uncertainty in these mass fluxes, a series of filters were applied to GRACE gravity information in a 1° geodesic grid domain. Those filters include (1) mascon geodesic grid correction, (2) Glacial Isostatic Adjustment (GIA) correction, (3) Degree-1 coefficients (Geocenter) corrections and (4) C20 (degree 2 order 0) replacement. The final total water storage change is obtained by subtracting the mean from 2003 to 2009. Please note that GRACE measures the storage anomaly at approximately monthly intervals, but it does not measure total quantity of water stored. GRACE storage anomalies were available monthly from April 2002 to June 2017 at the time of this analysis with a spatial resolution of 1°. Given the relatively low spatial resolution, Scalon *et al*.^19^ suggested to use GRACE only for watersheds which have areas greater than 100,000 km^2^ (The area of the UCRB is approximately 280,000 km^2^). Uncertainty analysis for CSR RL06 is not available yet, however, uncertainty value suggested for the RL05 version is roughly 2 cm^73^.

Four stations from the Community Collaborative Rain, Hail and Snow Network (CoCoRaHS) provide potential ET estimates. Additionally, the AmeriFlux station at Niwot Ridge, CO (US-NR1^74,75^) provides latent heat observations (which can be translated directly to ET by dividing to a unit of latent heat of evaporation of water 2256 kJ/kg). While ET stations are scarce in the UCRB, because of the diversity in their locations and temporal coverage, we feel that those observations still play a crucial role in the simulation evaluation. First is the range of elevation and land cover that those stations represent: two high elevation stations located at Niwot Ridge, CO (3050 m) and Crested Butte, CO (2912 m), one moderate elevation station located at Carbondale, CO (1887 m) and two low altitude stations located at Grand Junction, CO (1428 m) and Castle Valley, UT (1464 m). With respect to land cover, stations at Carbondale, CO and Crested Butte, CO are located in evergreen forest; stations at Niwot Ridge, CO and Grand Junction, CO are located in deciduous forest; the station at Castle Valley, UT is located in shrubland. We compared ParFlow simulations with CoCoRaHs data from June 2012 to present and AmeriFlux data from 1999 to present, respectively.

In addition to ET estimated from stations, we also used remotely sensed ET from Simplified Energy Balance (SSEBop) MODIS product to compare with simulated ET. The SSEBop model provides daily and 1-km ET estimations for the whole UCRB from 2000 to the end of the validation period and has been shown to be reliable in various regions^76,77^. Senay *et al*.^78^ simulates ET in SSEBop by using pre-defined hot and cold boundary conditions. Each pixel is assigned a hot and cold boundary values based on maximum air temperature and differential temperature. Based on land surface temperature (K) obtained from MODIS images, ET fraction is computed and then multiplied with a short grass reference (mm.d^−1^) and a scaling coefficient to produce final ET^79^.

Lastly, ground temperature data from the National Oceanic and Atmospheric Administration (NOAA) Regional Climate Center (RCC) was used for comparison. The NOAA RCC data consist of observations compiled from the Global Historical Climatology Network (GHCN^80^) database, and other federal and regional agencies. There are a total of 490 stations that monitor temperature. These stations are well distributed over the UCRB (green diamonds in Fig. 1).

Two of the remotely sensed products used for comparison, namely, MODIS-SCA and GRACE, are downscaled to match with the dataset’s spatial resolution of 1-km and geographic projection (specified at Table 1). Specifically, MODIS-SCA and GRACE data were downscaled from 5-km and 100-km, respectively, to 1-km using the Nearest Neighbor algorithm.

**Table 1:** Descriptive characteristics of the ParFlow output dataset.

Characteristic	Variables
Model outputs	Pressure; Saturation; CLM output
Data type	ParFlow Binary data
Data format	.pfb
Projection	‘+proj = lcc +lat_1 = 30 +lat_2 = 60 +lat_0 = 40.00000762944445 +lon_0 = −97 +x_0 = 0 +y_0 = 0 +a = 6370000 +b = 6370000 +units = m +no_defs’
Spatial Coverage	−427000.645–468000.345; −1315000.309–708000.309
Spatial Resolution	1 km × 1 km
Temporal Coverage	October 1, 1982 to September 31, 2019
Temporal Resolution	1 hour

### Evaluation metrics

For timeseries data, we primarily used two metrics to evaluate model performance, Spearman’s Rho and Total Absolute Relative Bias. As explained in Maxwell and Condon^38^, plotting these two metrics against one another produces a figure that will concisely describe a model’s ability to reproduce appropriate timing and magnitude of flows. We hereafter refer to this type of figure as a Condon Diagram. Spearman’s Rho was used to assess the differences in the simulated and observed variables timing while the relative bias measures differences in their volumes. If simulations are closed to observation, we expect high Speaman’s Rho value and low relative bias value. Spearman’s Rho is computed as:

1 $$srho=1-\frac{6{\sum }_{i=1}^{n}{d}_{i}^{2}}{n({n}^{2}-1)}$$

where *d*_*i*_ is the difference in the independent ranking for the simulated and observed values at *i* time step, *n* is the number of values in each time series. The Total Absolute Relative Bias is calculated as:

2 $$bias=\frac{\left|{\sum }_{i=1}^{n}{S}_{i}-{\sum }_{i=1}^{n}{O}_{i}\right|}{{\sum }_{i=1}^{n}{O}_{i}}$$

where *S* and *O* are simulated and observed timeseries, respectively, and *n* is the number of values in each time series.

Additionally, we used the Kling-Gupta Efficiency (KGE^81,82^) to evaluate the streamflow performance. The KGE coefficient is proposed by Gupta *et al*.^81^ to achieve a more balanced evaluation of simulated mean flow, flow variability and daily correlation than the traditional Nash-Sutcliffe efficiency (NSE^83^)^84,85^.

For spatial data, we used two categorical validation indices, namely, Probability of Detection and False Alarm Ratio:

3 $$POD=\frac{Hit}{Hit+Miss}$$

4 $$FAR=\frac{False}{Hit+False}$$

where *Hit* is grid where both simulated and observed events occurred; *Miss* is grid cell where the observed event occurred but the simulated one did not; *False* is a grid cell where the simulated event occurred but the observed one did not.

## Data Records

The dataset is available to the public through an unrestricted data repository hosted by CyVerse^86^. All the inputs for the simulation are included, namely, NLDAS (Table 2), related hydrologic and land-surface variables (Table 3) and a TCL script that contains the complete list of ParFlow input keys for the simulation.

**Table 2:** Hourly NLDAS inputs for the simulation.

Variable	Abbreviation	Unit	Spatial resolution
Visible or short-wave radiation	DSWR	W/m^2^	1 km
Long wave radiation	DLWR	W/m^2^	1 km
Precipitation	APCP	mm/s	1 km
Air Temperature	Temp	K	1 km
East-West wind speed	UGRD	m/s	1 km
South-North wind speed	VGRD	m/s	1 km
Atmospheric pressure	Press	pa	1 km
Specific humidity	SPFH	kg/kg	1 km

**Table 3:** Files that contain the model input parameters for the simulation.

File	Description
UpperCO.slope[x/y].rivth1500.pfb	Topographic slopes in x and y directions
UpperCO_init_press_1982.pfb	Initial pressure after the spin up process
UpperCO_IndicatorFile_v2.pfb	3-D indicator file of different soil, geology and bedrock types
UpperCO_v2.pfsol	3-D solid file of the model domain
Drv_clmin_v2.dat	Parameters of CLM model
Drv_vegm_v2.UC.dat	Vegetation type, cartesian coordinates for each grid of the domain
Drv_vegp.dat	Vegetation parameters for the IGBP classification

For each WY of simulation time, there are three output files per hourly timestep, namely, pressure, saturation and CLM output.

The hourly outputs shown in Table 4 and inputs shown in Table 2 were averaged into monthly variables for comparison to observations. Some additional quantities were calculated from ParFlow outputs (e.g. water table depth). These variables are listed in Table 5.

**Table 4:** Hourly ParFlow-CLM output variables and units.

Variable	Unit	Files
Pressure Head at every grid cell (3D)	[m]	ParFlow Pressure
Saturation at every grid cell (3D)	[m^3^/m^3^]	ParFlow Saturation
total latent heat flux	[W/m^2^]	CLM layer 0
total upward LW radiation	[W/m^2^]	CLM layer 1
total sensible heat flux	[W/m^2^]	CLM layer 2
ground heat flux	[W/m^2^]	CLM layer 3
net veg. evaporation and transpiration and soil evaporation	[mm/s]	CLM layer 4
ground evaporation	[mm/s]	CLM layer 5
soil evaporation	[mm/s]	CLM layer 6
vegetation evaporation (canopy) and transpiration	[mm/s]	CLM layer 7
transpiration	[mm/s]	CLM layer 8
infiltration flux	[mm/s]	CLM layer 9
SWE	[mm]	CLM layer 10
ground temperature	[K]	CLM layer 11
irrigation flux	[na]	CLM layer 12
Soil temperature layer 1	[K]	CLM layer 13
Soil temperature layer 2	[K]	CLM layer 14
Soil temperature layer 3	[K]	CLM layer 15
Soil temperature layer 4	[K]	CLM layer 16

**Table 5:** Monthly variables derived from the ParFlow output/input datasets.

Variables	Unit	Source
Water Ponding Depth	m	ParFlow Pressure output
Water Table Depth	m	ParFlow Pressure output
Snow Water Equivalent	mm/month	ParFlow CLM output
Total Water Storage	mm/month	ParFlow Pressure and Saturation output
Evapotranspiration	mm/day	ParFlow CLM output
Ground Temperature	K	ParFlow CLM output
Precipitation	mm/day	NLDAS Input
Soil Moisture	—	ParFlow Saturation output and static Porosity Input

ParFlow grid data is stored in a ParFlow binary file format (.pfb) which is written as BIG ENDIAN binary bit ordering. More information about ParFlow binary file format can be found in the ParFlow manual (https://github.com/parflow/parflow/blob/master/parflow-manual.pdf). Developed modules that read the ParFlow binary files can be found at https://github.com/parflow/parflow/tree/master/pftools/prepostproc and https://github.com/hydroframe/parflowio.

## Technical Validation

In this section we compared simulated water and energy fluxes to a wide range of datasets for the UCRB from 1983 to 2019, including station measurements and modeled outputs to validate the fidelity of the simulation and prioritize for future model improvement. The available station measurements were streamflow, water table depth (WTD), snowpack, water storage and evapotranspiration (Fig. 1). The available modeled outputs were Snow Cover Area, Total Water Storage Anomalies, and evapotranspiration.

### Streamflow comparisons

We compared simulated and observed monthly streamflow for eight representative stations within the UCRB (Fig. 2). Streamflow varied over two orders of magnitude with peak flow from around 2000 (ft^3^/s) at East River at Almont station to around 40,000 (ft^3^/s) at Colorado River near Cisco station and at Green River at Green River station. Simulated streamflow reflected accurately wet (1983–1985, 1993–1996, 2008–2010) and dry (1989–1992, 2002–2004, 2011–2013,2018) periods. In general, simulated flows matched observed ones well with average relative bias and Spearman’s Rho of 0.15 and 0.51, respectively.

The simulated and observed streamflow were in good agreement at Colorado River near Cisco station and at Green River at Green River station with low relative bias of 0.4 and 0.35 and high Spearman’s Rho of 0.52 and 0.73, respectively. Simulated flows at stations from the San Juan River had moderate bias between 1989 and 1992, but the bias reduced later. In the drought years between 1989 and 1992, we found NLDAS indicated more snow than one measured from SNOTEL and snow melt later than from SNOTEL, this snow component in NLDAS caused the moderate bias in streamflow simulation.

**Fig. 3:**
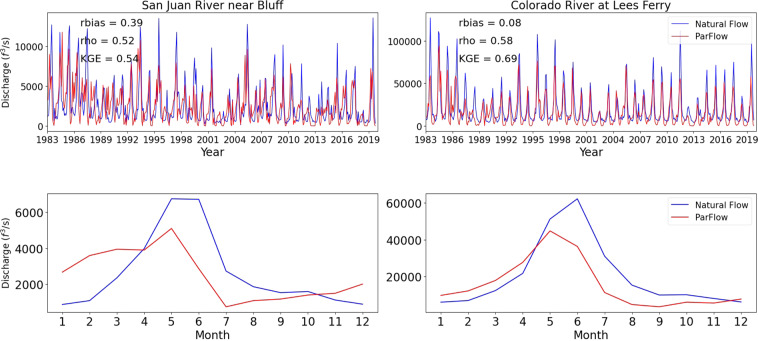
Natural flow comparison for two stations that are affected by upstream dams: San Juan River near Bluff and Colorado River at Lees Ferry. Streamflow predicted by ParFlow is shown using the red line while streamflow predicted by the natural flow model is shown in blue. Two panels above indicate monthly streamflow and two panels bellow indicate mean monthly for each month during the simulation period.

We found some discrepancies between streamflow simulations and observations in the station San Juan River near Bluff which could be attributed to anthropogenic activities. Model results were also compared to the Bureau of Reclamation natural flow dataset. Figure 3 shows examples of streamflow performances for two stations that are affected by upstream dams: San Juan River near Bluff and Colorado River at Lees Ferry. Overall, the ParFlow results shown in Fig. 3 are in good agreement with the naturalized flows. In the Colorado River at Lees Ferry station, the natural flow and ParFlow showed similar peaks and low streamflow bias across the comparison period. The mean monthly hydrograph comparisons highlighted a good correlation in seasonal cycles between the ParFlow results and the naturalized flows (Fig. 3). However, the ParFlow results were lower than the naturalized flows during summer and early winter in both stations (Fig. 3).

**Fig. 4:**
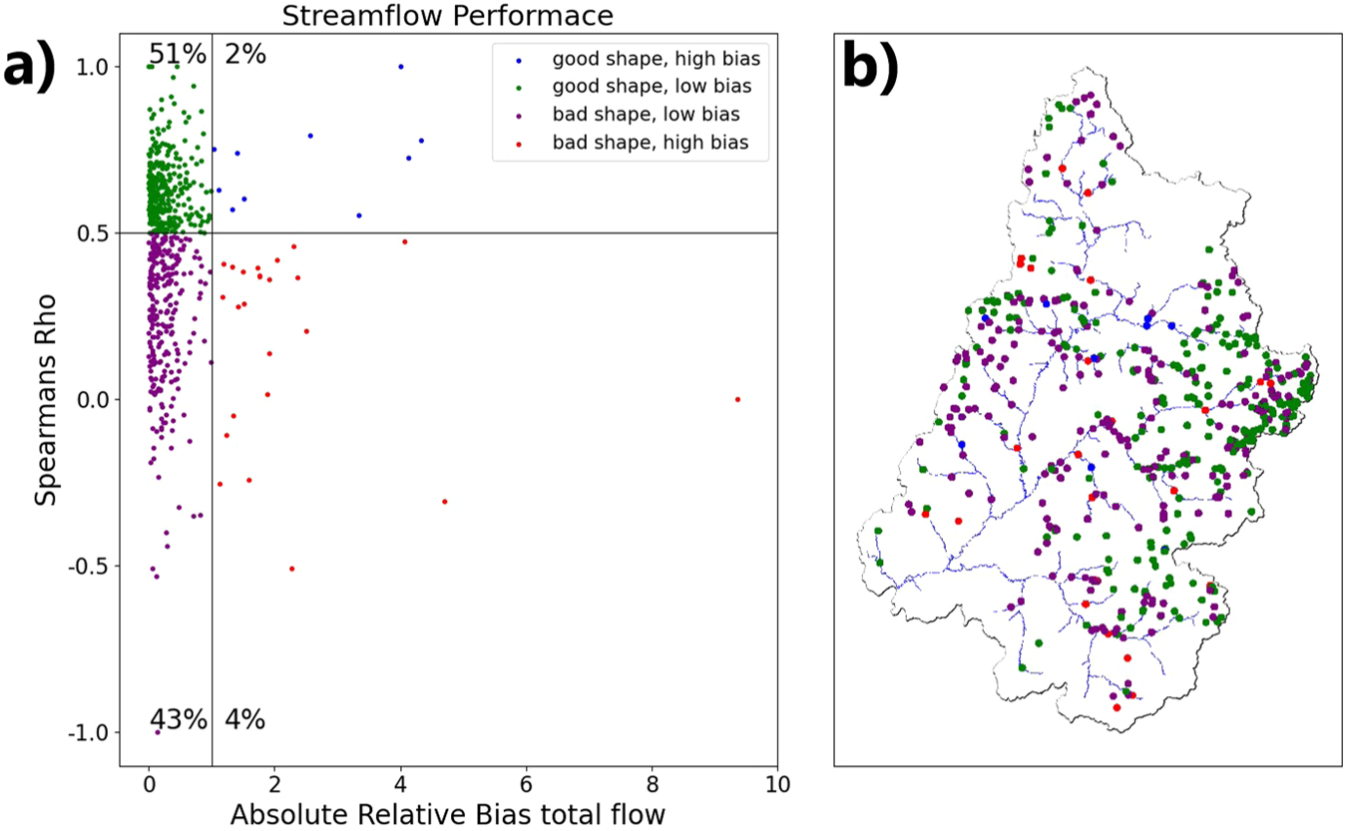
(**a**) The Condon-diagram streamflow performance plot, (**b**) the performance category of each gage within the UCRB domain.

Streamflow performance using Spearman’s Rho and relative bias is shown in Fig. 4 for all stations inside the UCRB. Stations were considered to have a good shape (i.e. matching temporal pattern) when their Spearman’s Rho values are greater than 0.5 and bad shape when their Spearman’s Rho values were smaller than 0.5. Likewise, stations were considered as low bias if their relative biases were smaller than 1 and high bias if their relative biases were greater than 1. Hence, there were four types of streamflow performance: (1) stations with good shape and low bias – green stations; (2) stations with good shape and high bias – blue stations; (3) stations with bad shape and low bias – purple stations; (4) stations with bad shape and high bias – red stations.

Despite the complex terrain of the UCRB, most of the stations (94%) had relative bias lower than 1. The majority of stations (52%) had both low bias and good shape (i.e. relative bias smaller than 1 and Spearman’s Rho greater than 0.5), most of them located in the upstream part of rivers originating from the Rocky Mountains, namely, the Gunnison, Yampa and San Juan. Simulated flows matched with observed ones along the Green River (Figs. 2 and 4). Stations with bad shape generally fall into two categories: (1) impacted by anthropogenic activities (e.g. stations along the Colorado River); (2) located in relatively small streams (e.g. western tributaries of the Green River or tributaries of the San Juan River). The results agreed well with previous ParFlow streamflow evaluations of the UCRB^38,87^.

### Water table depth comparisons

**Fig. 5:**
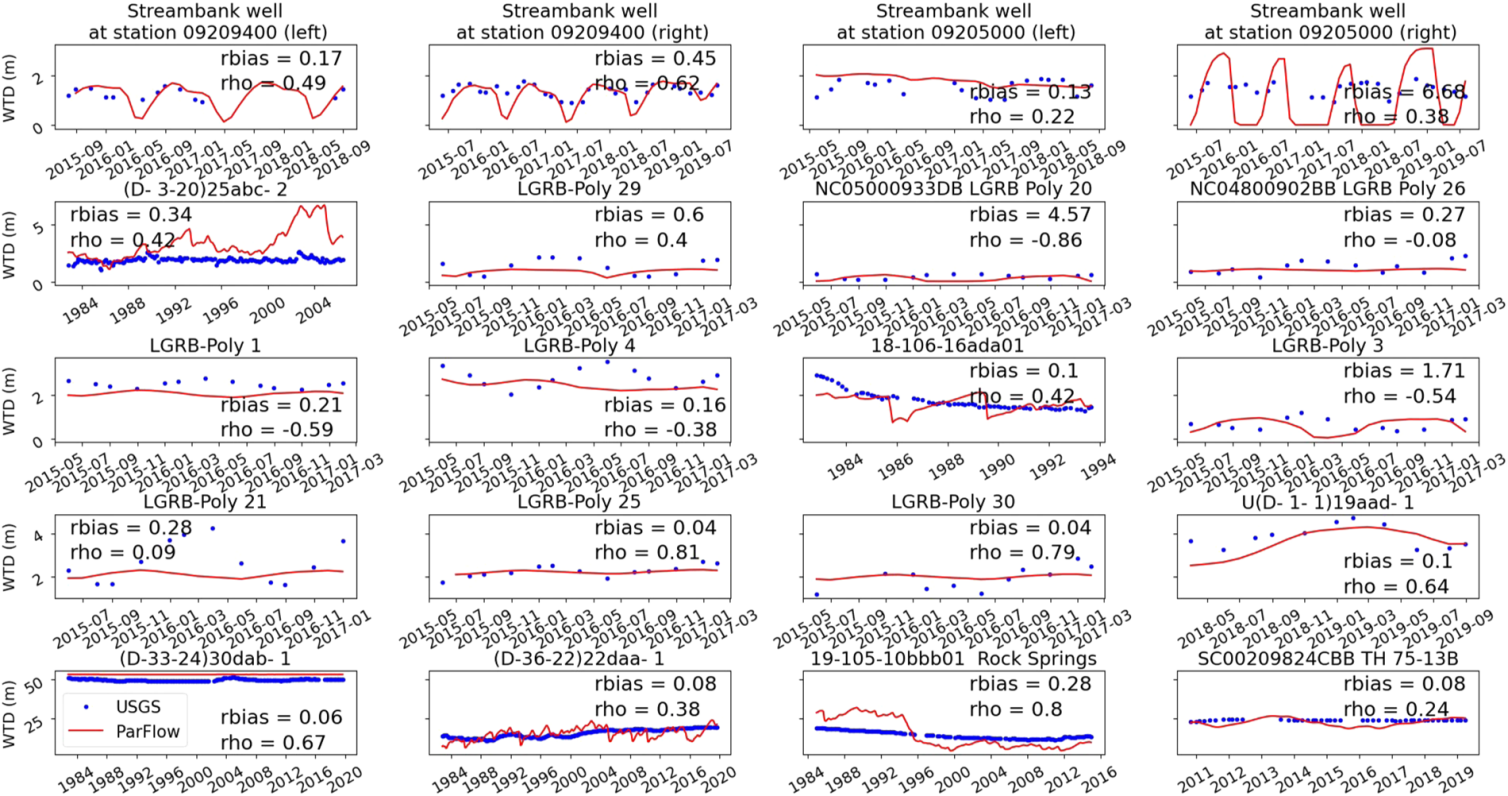
Time series plots of predicted and observed water table depths for available USGS wells within the basin.

A representative sample of wells is taken for comparison between observed and simulated WTD (Fig. 5). ParFlow-CLM accurately simulated both timing and magnitude for most of the wells, however there were some points of discrepancy.

Overall, both streambank and deep wells had good agreements between simulations and observations with the relative bias for 14 wells out of 24 wells was smaller than 0.3. Simulated WTDs at the Lower Gunnison River Basin (LGRB) matched close to observations in mean depth and temporal variability. Similarly, simulated streambank wells (e.g. at station 09205000 (left) and at station 09209400 (left and right) showed a good agreement with observed WTD. An exception to this close agreement was the well at the right bank of station 09205000 where simulated WTD showed much greater seasonal cycles than observed.

Deep wells with WTD of around 20 m such as 19–105-10bbb01 Rock Springs (near Rock Springs, WY) and (D-36-22)22daa-1 (near Blanding, UT), showed decreasing and increasing trends, respectively, over time and simulated WTD shows similar trends. With wells that are close to the maximum WTD of the domain (e.g. (D-33-24)30dab-1), simulated WTD did not reflect the observed multi-year cycle in these locations. This bias was likely caused by the model spatial resolution. Deeper wells are often located in mountainous areas where the topography is complex. ParFlow-CLM’s 1-km resolution may not be sufficient to capture this type of behavior. Please note that all wells were mapped to the closest grid cell center with no further adjustment.

**Fig. 6:**
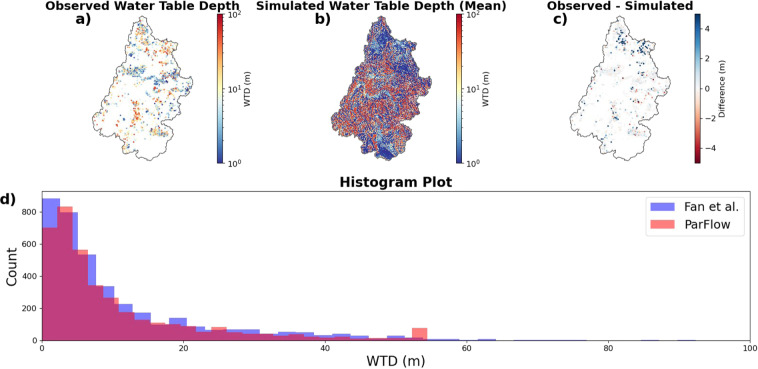
Spatial comparisons of water table depth averaged over the entire simulation. (**a**) Average WTD for USGS sites between 1927 and 2009 from Fan *et al*. (2013), (**b**) The average WTD from this simulation, (**c**) observed-simulated WTD, (**d**) histogram of simulated and observed WTD.

We also compared the average simulated WTD (Fig. 6b) with the average USGS WTD dataset compiled from Fan *et al*. 2013 (Fig. 6a). Most of the wells had WTD differences of less than 1 m compared to the simulations, and some deep wells (around 40-m depth) in the Green and San Juan sub basins were 2–4 m deeper than the simulated. The observed dataset had more shallow water tables than simulated (Fig. 6c,d) and simulated WTD had more points which reach the maximum depth of 52-m (Fig. 6d). The output WTD was on average over one square kilometer (model grid spatial resolution). Most of the “max depth” points were in the mountainous areas where the average output WTD was often dominated by WTD that is close to 52-m. This often led to overestimation of the depth (i.e. WTDs are deeper than in observation wells). However, the WTD average for most of the well was consistent with the observations with nearly 2,650 wells (70%) having absolute differences smaller than 0.5 m. Lastly, the average of absolute difference in depth for all the wells was 1.042 m.

### Snow covered extent comparison

**Fig. 7:**
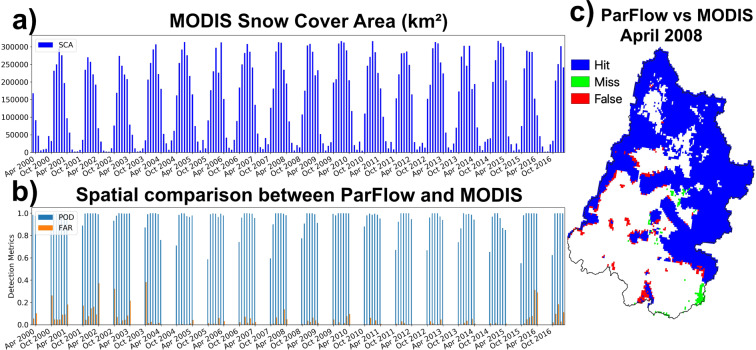
(**a**) MODIS monthly snow cover area (SCA) for the Upper Colorado River Basin from 2000 to 2016. (**b**) Spatial comparison between ParFlow and MODIS SCA using two categorical validations, namely, Probability of Detection and False Alarm Ratio. (**c**) An example of spatial comparison between ParFlow SCA and MODIS SCA.

We compared the simulated SCA to one obtained from the cloud-free MODIS product (Fig. 7a). Model outputs were upscaled and averaged to match with the cloud-free product’s spatial and temporal resolutions. Simulated SCA had consistent high Probability of Detection (average 0.82) throughout the period of 17 year (Fig. 7b). False Alarm Ratio was often high (around 0.4) during accumulating and melting months (i.e. October and April, respectively). During winter, simulated SCA accurately reflected the dynamic of SCA with almost 100% snow pixel captured and below 5% overestimated snow pixels. For typically dry years such as 2001, 2002 and 2016, ParFlow-CLM tended to produce slightly more SCA.

Figure 7c shows a snapshot of comparison between simulated and cloud-free MODIS SCA in a melting month of April 2008. Both SCAs agreed well in the Green and Gunnison river basins. Cloud-free MODIS SCA indicated that snow had already melted in various places along the Colorado River and Northern Arizona (lower left of the UCRB) while it was contrasting in the simulated SCA. Lastly, the model missed some snowpack in Northern New Mexico (lower right of the UCRB). A detailed discussion regarding sources of bias in snow simulation is covered in the next section.

### Snow water equivalent comparisons

**Fig. 8:**
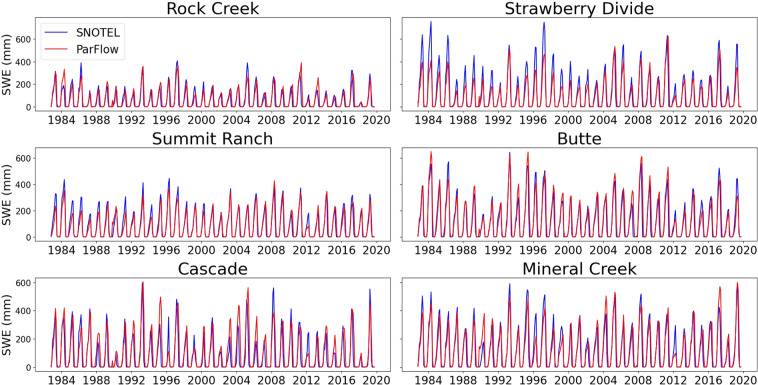
Time series plots of predicted and observed (SNOTEL) SWE over the simulation time period for six SNOTEL stations within the UCRB.

**Fig. 9:**
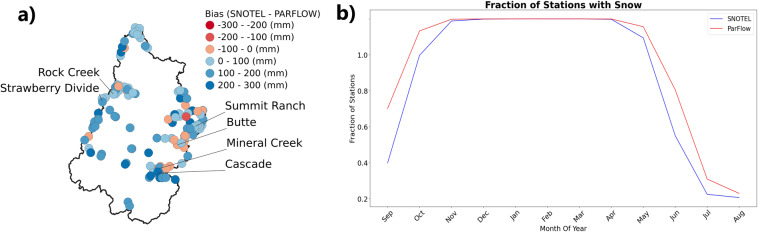
(**a**) Predicted-observed bias for all the SNOTEL stations within the UCRB, and (**b**) plot of the fraction of SNOTEL stations with snow compared to the equivalent model simulated output.

All SWE stations were mapped to the closest grid cell center with no adjustments made to scale point value to grid block or to adjust for differences between the station’s elevation and the mean grid cell elevations. Comparison results for six representative stations from Green, Gunnison and San Juan basins are shown in Fig. 8. While Rock Creek and Strawberry Divide stations are in Green basin, Summit Ranch and Butte stations are in Gunnison basin, Cascade and Mineral Creek are in San Juan basin (Fig. 9). Elevations of these stations ranges from the lowest of 2403 m (Rock Creek) to the highest of 3100 m (Butte). In general, simulated and observed SWE agreed on peak timing and magnitude trend over the period. Simulated SWE accurately reflected drought periods (i.e. 1988–1990, 2002–2004, 2012–2014 and 2018) and wet periods (1983–1985, 1993–1995 and 2008–2010).

However, before 2003, simulated SWE was systematically lower than observed in all stations. This dry bias was worst in the Strawberry Divide station, where simulated peak monthly SWE was often 200 mm lower than the observed. A potential source of bias was directly linked to the NLDAS dataset. Earlier version of NLDAS used in this simulation from WY 1983 to WY 2002 has been shown to have SWE bias from: (1) dry bias in annual precipitation, (2) air temperatures are systematically lower in winter and higher in spring, (3) coarse spatial resolution of 1/8° ^49,50^. For simulation from WY 2003, we used a newer version of NLDAS which was greatly enhanced in precipitation and temperature estimations^88^. The dry SWE bias was much alleviated overall although the bias persisted during some drought years of 2012 and 2013.

Figure 9 shows an interesting contradiction. While simulated maximum annual SWE was lower than observed SWE for most of the stations (Fig. 9a), when doing monthly average by year, ParFlow-CLM simulated earlier snow accumulation and later snow melt than observations (Fig. 9b). In the Green basin, maximum annual SWEs were often lower by 100 m than observations. In contrast, basins in higher altitude such as Gunnison and San Juan had both underestimated (smaller by 200 mm) and overestimated (greater by 100 mm) stations.

Beside bias in forcing, there always existed an inconsistency when comparing point measurements with the model 1-km grid. The model grid resolution could smooth out the dynamic changes of snowpack in high altitude regions. Figure 9b shows a reverse pattern of fraction of station with snow between SNOTEL and ParFlow-CLM that Maxwell and Condon (2016) found in water year 1985. The reverse pattern could be a result of replacing forcing inputs in the second half of the decadal simulation.

### GRACE terrestrial water storage comparison

**Fig. 10:**
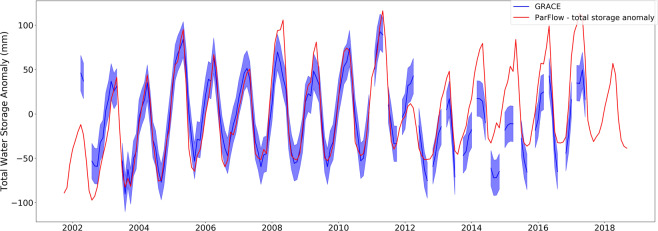
Time series plot of the total water storage anomaly from the model simulations and the GRACE estimates.

Changes in water storage from GRACE were compared with the ParFlow-CLM simulation from 2002 and 2017 (Fig. 10). Water storage change from GRACE was obtained by subtracting the water storage mean from 2004 to 2009. We also did the same process with outputs from ParFlow-CLM to ensure the consistency in comparison. Thus, we compared between the products basin-wide.

Two products agreed well from 2003 to 2011. Drought years of 2003 and 2004 were accurately reflected as well as wet years of 2005, 2008 and 2011. During periods of data discrepancy from GRACE (i.e. 2002 and from 2011 to 2017), GRACE showed much lower water storage anomaly than ParFlow-CLM.

### ET comparisons

**Fig. 11:**
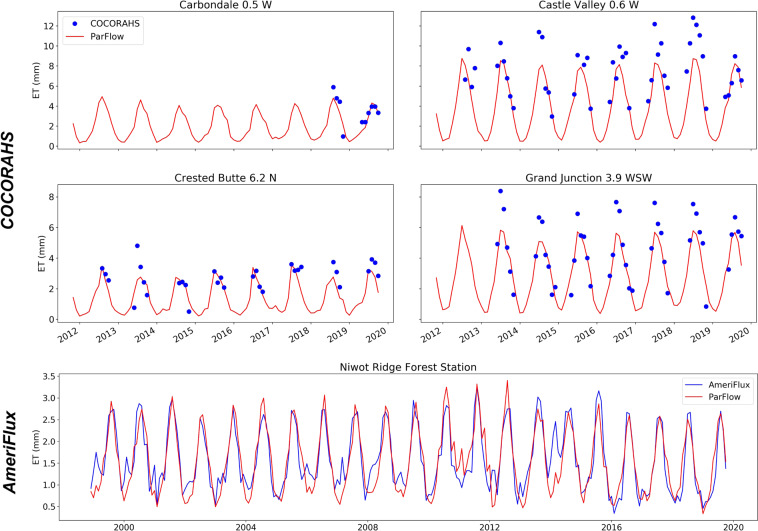
Comparison between simulated ET and estimates of ET based on observations. The upper four panels are potential evaporation from the COCORAHS network, the bottom panel actual ET from the Niwot Ridge eddy covariance station.

Direct observations of ET and/or latent heat were from two sources: (1) AmeriFlux network (one station, data from 1999) and (2) COCORAHS (four stations, data from 2012). Stations from COCORAHS measure potential evaporation. Measuring potential ET rather than actual ET by COCORAHS explained the systematic underestimation of simulated ET in months of June and July for Grand Junction and Crested Butte and in months of May, June and July for Castle Valley, as potential ET would be greater than actual ET. For other summer months, ParFlow-CLM simulated accurately with observations (Fig. 11).

The Niwot Ridge Forest station from the AmeriFlux network is located at 3050 m elevation in subalpine forest. Flux magnitude and trend from both simulated and observed ET were overall matching (Fig. 11). Niwot Ridge station showed small amounts of evaporation (around 1.5 mm) during winters of 1999, 2000, 2007, 2009 and 2014 while ParFlow-CLM did not show winter ET values.

**Fig. 12:**
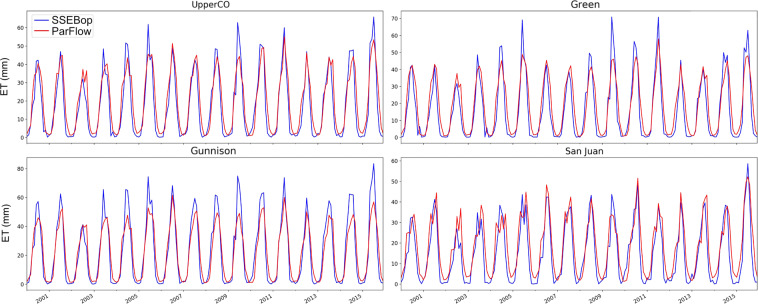
Comparisons between the SSEBop remote sensing product and model simulations for ET for the entire domain (UpperCO) and three major sub-basins.

When comparing with a remotely sensed product (SSEBop) over the entire basin, the simulated ET had lower ET peak than SSEBop in the Green, Gunnison and ultimately Upper Colorado River basins (Fig. 12). In the San Juan basin, the two ET estimates agreed well over the period. The SSEBop’s estimation approach is fundamentally different from the physical based modelling of ParFlow-CLM, and thus requires detailed comparisons of model specifications in order to examine the difference in ET.

### Land surface temperature comparison

**Fig. 13:**
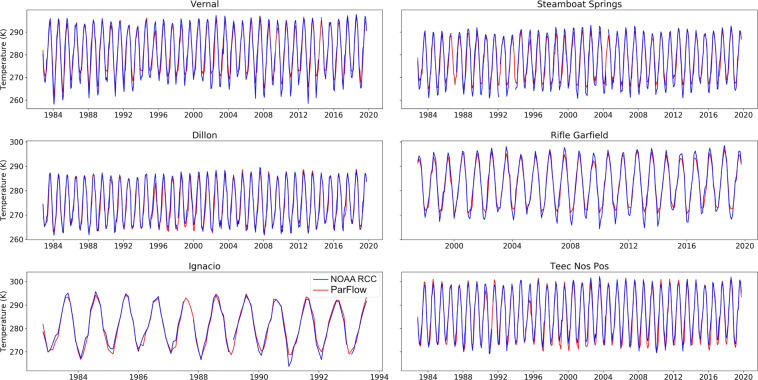
Land surface temperature comparison plots for model simulations and observations at six stations within the UCRB.

Simulated and observed ground temperature matched closely throughout the study period. Out of 490 NOAA RCC stations, 263 stations had Spearman’s Rho value greater than 0.95, 486 stations had relative bias smaller than 0.05. Figure 13 shows comparison for six representative stations for the Green, Gunnison and San Juan basins (Vernal and Steamboat Springs in Green, Dillon and Rifle Garfield in Gunnison, Ignacio and Teec Nos Pos in San Juan). We can see great matches between simulations and observations in different terrains and temperature ranges.

### Comparison summary

We compared seven variables output from the decadal simulation, (1) streamflow, (2) water table depth, (3) snow cover area, (4) snow water equivalent, (5) actual evapotranspiration, (6) total storage water anomalies and (7) ground temperature, to observations and data products. In general, this comparison demonstrates good agreements between model and observations and data products.

A summary of comparisons is given in Tables 6, 7. The average relative bias for streamflow, WTD, SWE, ET and temperature was 0.123. For this pre-development simulation, more than half of the stream gauges (53%) had good timing correlation (Spearman’s Rho > 0.5) between simulated and observed streamflow. This simulation accurately captured the mean WTD with 70% of the wells having absolute difference in mean WTD smaller than 0.5 m. Other land surface variables such as: SWE, ET, TWSA and ground temperature also showed a good match to observations and had an average relative bias of 0.078 and an average Spearman’s Rho of 0.838.

**Table 6:** Comparison summary for point observations.

Point Observation	Variable Measured (unit)	Average Monthly Relative Bias	Average Monthly Spearman’s Rho	Average Depth Difference	Number of Stations
USGS stream gauges	Discharge (ft^3^/s)	0.043	0.460	—	602
Bureau of Reclamation’s natural flow	Discharge (ft^3^/s)	0.05	0.68		20
USGS wells	WTD (m)	0.356	0.432	—	36
Fan *et al*. Compile WTD	WTD (m)	—	—	1.042	3865
SNOTEL	SWE (mm)	0.213	0.840	—	133
CoCoRaHS	Potential ET (mm/d)	0.123	0.667	—	4
AmeriFlux	Actual ET (mm/d)	0.001	0.854	—	1
NOAA RCC	Temperature (K)	0.001	0.958	—	490

**Table 7:** Comparison summary for remotely sensed products.

Remotely sensed Product	Variable Measured (unit)	Temporal Coverage	Average Monthly Relative Bias	Average Monthly Spearman’s Rho	Average Monthly POD	Average Monthly FAR
Cloud-free MODIS SCA	SCA (km2)	March 2000–February 2017	—	—	0.737	0.193
MODIS SSEBop	Actual ET (mm/month)	March 2000–December 2015	0.050	0.866	—	—
CSR GRACE RL06 Mascon	Total water storage change (mm)	April 2002–June 2017	0.069	0.863	—	—

Sources of bias in simulated streamflow and WTD were due to four reasons: (1) lateral and vertical resolutions; (2) water management operations; (3) bias in meteorological forcing data; (4) uncertainties in subsurface properties.

## Usage Notes

In the CyVerse repository, beside ParFlow output files, we also provided necessary input files to reproduce the simulation. Specifically, we included (1) NLDAS data; (2) ParFlow and CLM parameter files (e.g. vegetation parameter file, subsurface indicator file and topographic information files); (3) A tcl script for running the simulation. More information can be found in the repository readme file.

Interested users are encouraged to use the newly developed parflowio tool (https://github.com/hydroframe/parflowio) to work with.pfb format files.

While it was shown that anthropogenic activities reflected in USGS streamflow and WTD still contributed to the comparison bias, the results are encouraging with most of the gauges and wells having low relative bias and high Spearman’s Rho scores. As Maxwell and Condon^38^ indicated that the ParFlow simulation platform is evolving with more refined surface^89^ and subsurface and more anthropogenic activities coupled, the model formulation will be improved.

The dataset produced in this study is useful in hydrological studies due to its high spatial and temporal resolutions and validated accuracy. Moreover, having more consistent data about the groundwater dynamic, one can study its impact on the full water cycle of the UCRB and possibly other river basins. We hope the dataset will be used for a wide range of stakeholders from decision makers to ecologic scientists.

## Data Availability

These simulations were conducted using ParFlow version 3.6.0 (https://github.com/parflow/parflow/tree/v3.6.0/). The data processing step was done using Python3.5 programming language with necessary toolboxes including NumPy (https://numpy.org/), the Geospatial Data Abstraction Library (GDAL; https://gdal.org/) and the Python Data Analysis Library (PANDAS; https://pandas.pydata.org/).
